# ImageJ assessment of double-frequency YAG laser efficacy in treatment of mild lower eyelid ectropion with punctal eversion

**DOI:** 10.1007/s10792-022-02590-1

**Published:** 2022-11-28

**Authors:** Moustafa Salamah, Hesham A. Enany, Mohammed A. Hegab, Hani A. Albialy

**Affiliations:** Department of Ophthalmology, Faculty of Medicine, Zagazig University, Zagazig, Egypt

**Keywords:** Punctal eversion, Medial ectropion, Tear film, Double-frequency YAG laser, ImageJ

## Abstract

**Purpose:**

To describe a simple and minimally invasive technique using double-frequency YAG laser for correction of mild medial lower eyelid ectropion with punctal eversion and assess the efficacy of the technique by measuring the tear film meniscus pre- and post-laser treatment using imageJ software.

**Methods:**

This study included 23 eyes of 19 patients with lower eyelid ectropion with punctal eversion. All patients were treated by double-frequency YAG laser applied to lower medial conjunctiva. Tear film thickness was assessed using imageJ software pre- and post-argon laser treatment.

**Results:**

There was a highly significant change detected after argon laser treatment as regarding the mean of the height of tear film which was found to be significantly lower after argon laser treatment compared to before it (81.1 pixels versus 193.1 pixels, respectively) (*P* < 0.001).

**Conclusion:**

Double-frequency YAG laser applied to lower medial palpebral conjunctiva is a simple, easy, safe, and effective procedure which can be used as a solo treatment in early cases of punctal eversion with no or mild medial canthal tendon laxity.

## Introduction

Involutional (Senile) medial ectropion of the lower eyelid is an anatomic malposition in which the medial aspect of the eyelid is rolled away from the globe. Occurrence of ectropion involves an interplay of different factors; the aging process results in atrophy of muscular and tendon structures with inferior retractor dehiscence, horizontal eyelid laxity, and disinsertion of fascial attachments between the anterior and posterior lamella [1, 2]. As a consequence of ectropion, the punctum becomes visible and everted causing various symptoms such as epiphora, ocular irritation, foreign body sensation, pain, corneal ulcers, and scarring. These symptoms force the patient to eye rubbing, which may further exacerbate the condition [3].

The most important factors in decision-making for surgical repair of involutional ectropion are the severity of the dysfunction and the degree of canthal tendon laxity. Surgical procedures aim at lid shortening either horizontal or vertical shortening to bring it in a good apposition with the globe. In cases of medial lid laxity, an inverting procedure to the medial part of the eyelid, with correction of any horizontal eyelid laxity obtained by full-thickness lid resection, medial canthal suture, or transconjunctival diamond excision in the medial part of the lid (the ‘lazy-T’ procedure) or the medial spindle procedure, both of which require excision of posterior lamella tissue [1, 4–8].

In medial ectropion, the close relation with the punctum and the canalicular system renders these surgical procedures more complicated. These procedures may sacrifice medial lower eyelid tissue or result in disruption of the lacrimal system [9].

For this fact, we described double-frequency YAG laser applied to the conjunctival surface of the medial aspect of the lower eyelid for correction of cases with mild senile medial ectropion with lower punctal eversion accompanied with no or mild medial canthal tendon laxity.

ImageJ is one of the most image analysis programs commonly used in the biological sciences. ImageJ software has been previously used in different subspecialties of ophthalmology; Bandlitz and Pult used it to measure tear film [10]. Simoes et al. used it to measure peripapillary choroidal thickness [11], while Rodrigues and co-authors used it to measure corneal incision architecture [12].

This study aimed to evaluate the use of double-frequency YAG laser as a simple technique for the treatment of cases of mild medial lower lid ectropion with punctal eversion and the use of imageJ software to assess the efficacy of the technique by measuring the tear film meniscus height pre- and post-argon laser treatment.

## Patients and methods

This prospective, interventional study included 23 eyes of 19 patients with mild medial lower eyelid ectropion with punctal eversion who underwent double-frequency YAG laser application on lower medial palpebral conjunctiva in the period from February 2018 to August 2020.

The technique, likely post-treatment results, and potential complications were explained to all patients. Written consent was obtained from all patients included permission to publish their photographs. This research was approved by the Institutional Review Board of the Alpha Vision Center and was adherent to the ethical principles outlined in the Declaration of Helsinki as amended in 2013.

### Preoperative assessment

All patients provided a full medical history and received a detailed ophthalmological examination.

**Fig. 1:**
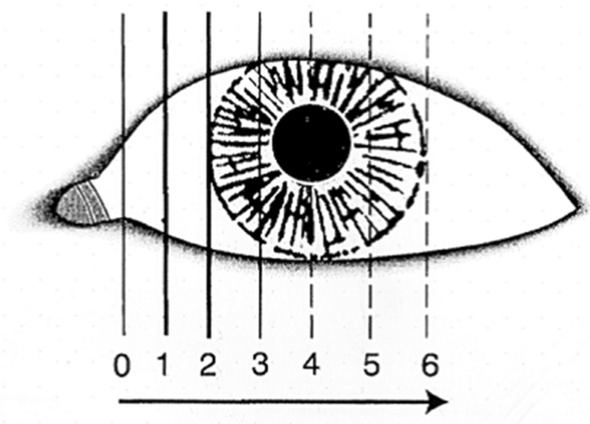
Lateral distraction test and grading of lower lid laxity

Patients were assessed for eyelid and punctual position, any associated medial canthal tendon (MCT) laxity, and accompanying symptoms. Lower eyelid laxity was evaluated using the lateral distraction test. A lateral distraction test is performed by displacing the medial canthal tendon laterally. The endpoint is the point at which the punctum stops on full lateral distraction and can no longer be pulled laterally with sustained traction and ranges from grade 1 to 6. [13] (Fig. 1).

Fluorescein dye disappearance test and syringing of lacrimal drainage system were done for all patients to exclude anatomical obstruction. Only patients with lateral distraction test grades ≤ 2 were included in this study. Patients with more advanced grades of medial canthal tendon laxity, previous lid surgery, lacrimal passage obstruction, severe dry eye, skin scarring, and blepharitis were also excluded from the study.

#### Technique and follow-up

Topical anesthesia benoxinate 0.4% eye drops were applied five times with two minutes apart. A corneal shield was applied for protection from laser light.

**Fig. 2:**
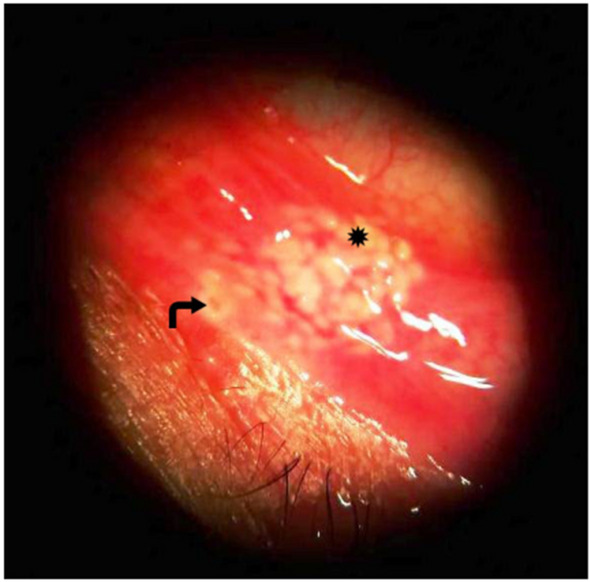
Post-argon laser; the curved arrow represent punctum and the astric represent laser mark on conjunctival surface

The following parameters were used with a German Zeiss double-frequency YAG Laser Visuals 532 s slit-lamp mounted green laser: 200–400 micron for the spot size, 0.3–0.5 s for the pulse duration, and 500–700 MW power. The patient was told to look upward. To make it easier to see the palpebral conjunctiva, the index finger was used to evert the medial portion of the lower eyelid. An end point of edge-to-edge multiple white laser burns with visible tissue contraction was created by applying a double-frequency YAG laser on the palpebral conjunctival surface of the medial aspect of the lower eyelid in an area of diamond-shaped pattern 5 mm in length, with the apex of the diamond 2 mm below the punctal opening (Fig. 2).

Primary outcome measures included measurement of the marginal tear film height by ImageJ software to assess the functional success. Secondary outcome measures included restoration of the normal lower punctual position to assess the anatomical success and improvement, or disappearance of patient’s complaints (epiphora or discomfort) as reported by patient satisfaction.

Antibiotic Gatifloxacin (Tymer) (Jamjoom Pharmaceuticals, Jeddah—Saudi Arabia) eye drops five times daily and paracetamol (Adol) (Gulf Pharmaceutical Industries- United Arab Emirates) 500 mg tablets three times daily were prescribed as a post-laser treatment for one week. We didn’t use any anti-inflammatory drugs as we believed these drugs may decrease the post-laser fibrotic effect on the conjunctiva.

The follow-up period was 6 months. Photographic documentation was carried out before laser treatment and at the end of the follow-up period. Standardization of pre- and post-laser treatment of tear meniscus photographs was assured by using the same type of fluorescein strip and the same application time of 10 s. The photographs were taken after 4 min after removal of fluorescein strips. We used the same slit lamp with the same magnification and the same working distance in each pre- and post-laser photographs.

**Fig. 3:**
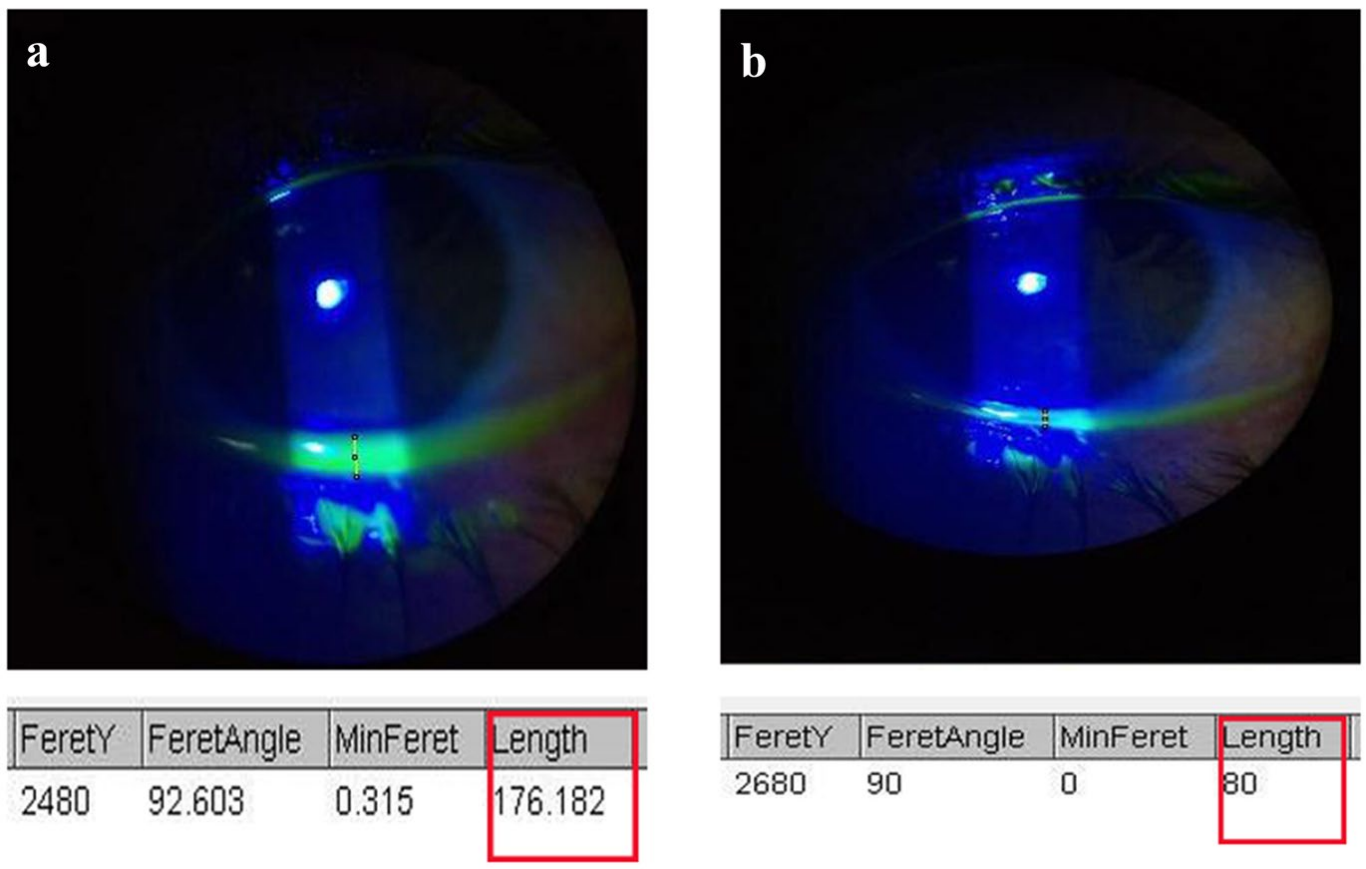
**a** Pre-argon laser treatment tear film thickness equal to 176 pixels. **b** 6-month post-argon laser treatment tear film thickness equal to 80 pixels

Analysis of preoperative and 6-month postoperative photographs was performed using ImageJ version 1.2.4 software to evaluate the height of tear film (Fig. 3). All patients were assessed pre- and post-laser treatment by a senior blinded investigator for the evaluation of correction of lower punctual position. All data were collected, tabulated, and statistically analyzed using SPSS version 20.

## Results

This prospective, interventional study was performed on 23 eyes of 19 patients (4 had bilateral lower lid punctal eversion) including 13 men and 6 women, with a mean age of 64.8 years (range 54–73).

Watery eye was the commonest complaint followed by ocular irritation.

Following laser application, all cases complained with temporary mild pain & discomfort that respond to paracetamol 500 mg tab, three times daily for the first postoperative 7 days.

**Fig. 4:**
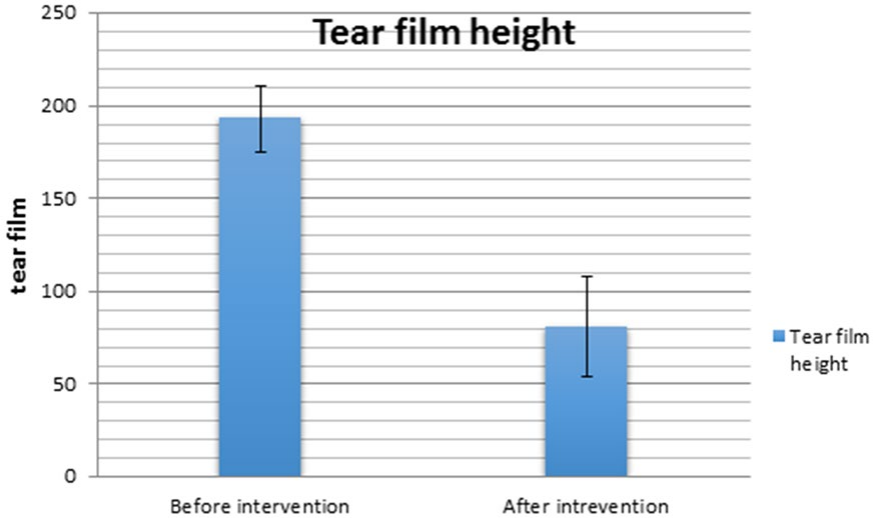
Bar chart showing the mean tear film height measurements pre- and 6 months post-argon laser therapy

There was a highly statistically significant difference between the preoperative and postoperative values of the height of the marginal tear film "using ImageJ software" which was found to be significantly lower after argon laser treatment compared to before it (mean 81.1 pixels versus mean 193.1 pixels respectively) (Table 1; Fig. 4).

**Table 1:** Tear film meniscus height measured in pixels pre-treatment, during and at the end of follow-up

Variable	Pre-treatment	One week post-double-frequency YAG laser treatment	4 weeks post-double-frequency YAG laser treatment	12 weeks post-double-frequency YAG laser treatment	24 weeks post-double-frequency YAG laser treatment
Tear film height in pixels	212.0	248.0	93.0	91.0	89.0
	196.0	213.0	61.0	56.0	54.0
	189.0	233.0	77.0	73.0	73.0
	185.0	198.0	62.0	56.0	56.0
	194.0	222.0	74.0	67.0	67.0
	176.0	184.0	56.0	54.0	53.0
	209.0	254.0	176.0	152.0	144.0
	216.0	262.0	69.0	66.0	64.0
	184.0	202.0	55.0	53.0	51.0
	191.0	198.0	104.0	99.0	98.0
	203.0	184.0	76.0	74.0	74.0
	169.0	195.0	81.0	78.0	77.0
	201.0	182.0	76.0	74.0	73.0
	183.0	188.0	102.0	93.0	91.0
	235.0	256.0	182.0	166.0	163.0
	179.0	193.0	69.0	68.0	69.0
	199.0	224.0	74.0	72.0	72.0
	192.0	176.0	81.0	76.0	74.0
	224.0	212.0	85.0	87.0	86.0
	176.0	152.0	92.0	88.0	80.0
	189.0	203.0	69.0	68.0	69.0
	157.0	132.0	89.0	88.0	87.0
	184.0	195.0	109.0	99.0	101.0
Mean tear film height in pixels	193.1	204.6	87.5	82.5	81.1

Three categories of post-laser punctum positions were identified: seen (both the anterior and posterior borders of the punctum were visible), partially seen (only the anterior border of the punctum was visible, but the posterior border was not), and not seen (neither anterior nor posterior borders were seen).

**Fig. 5:**
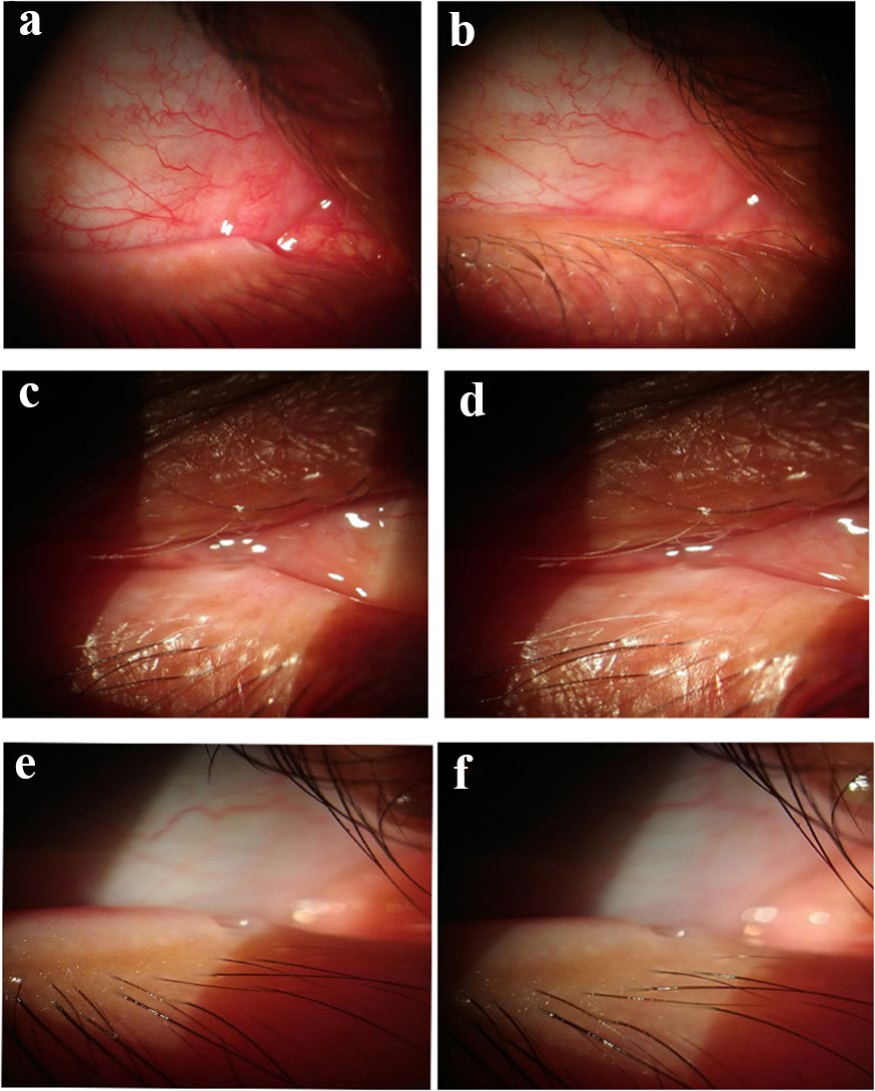
Case 1 **a** clearly seen punctum before argon laser application. **b** Punctum is not seen at the end of follow-up. Case 2 **c** clearly seen punctum before argon laser application. **d** partially seen punctum after at the end of follow-up. Case 3 **e** Clearly seen punctum before argon laser application. **f** Still seen punctum at the end of follow-up

As regards the anatomical success results, the punctum was not seen (return to the normal anatomical position) in 69.6% after the intervention compared to 0% before it with a highly statistically significant difference (Table 2; Fig. 5).

**Table 2:** Comparison of punctal visibility before and 6 months post-double-frequency YAG laser among the studied group (anatomical success):

Variable	Before intervention (*n* = 23)	6 months after intervention (*n* = 23)	*P* value
	*N* (%)	*N* (%)	
Punctum			
Seen	23 (100)	2 (8.7)	
Partially seen	0 (0)	5 (21.7)	< 0.001
Not seen	0 (0)	16 (69.6)	(HS)

Regarding patient satisfaction, 60.9% of the studied participants were highly satisfied with diminished or relieved symptoms (Table 3).

**Table 3:** Degree of patient’s satisfaction among the studied group:

Variable	6 months after intervention (*n* = 23)
	*N* (%)
Degree of satisfaction	
Not satisfied	3 (13)
Partially satisfied	6 (26.1)
Highly satisfied	14 (60.9)

Postoperatively, there was a highly significant correlation between different degrees of patient satisfaction and the height of tear film, which was found to be significantly lower among those who were highly satisfied compared to those with partial and no satisfaction (70.2 pixels versus 78.8 pixels and 136 pixels respectively).

By comparing percent reduction of tear film among the different degrees of satisfaction, it was found to be higher among those who were highly satisfied compared to those who were partially or not satisfied (63.1%, 57.9%, and 35.6% respectively) with a highly significant difference between them (Table 4).

**Table 4:** Correlation of height of tear film and different degrees of patient's satisfaction among the studied group:

Variable	Not satisfied (*n* = 3)	Partially satisfied (*n* = 6)	Highly satisfied (*n* = 14)	*P* value
Tear film before intervention				
Mean ± SD	209.3 ± 25.5	190.3 ± 20	190.1 ± 14.8	0.255
Range	184–235	157–212	169–224	(NS)
Tear film 6 months after intervention				
Mean ± SD	136 ± 31.7	78.8 ± 9.30	70.2 ± 14.1	< 0.00
Range	101–163	67–89	51–98	1(HS)
% Reduction				
Mean ± SD	35.6% ± 8.2	57.9% ± 8.2	63.1% ± 7.6	< 0.001
Range	30.6–45.1	44.5–65.4	48.6–72.4	(HS)

*NS* nonsignificant, *HS* highly significant

No complications were recorded in this study except for two patients with mild early red eye following double frequency YAG laser which resolved within two weeks.

## Discussion

Ectropion is a common disorder encountered in the clinic, and its early and adequate management is of utmost importance. Repair of involutional ectropion remains a challenge in oculoplastic surgery. Depending on its etiology and the underlying pathology, a variety of surgical procedures were described [14]. The close position of MCT and orbicularis muscle with the canaliculi and lacrimal apparatus is an alarming point for surgeons in ectropion repair, as complex surgical procedures with medial eyelid resection may sacrifice medial lower eyelid tissue with disruption of the lacrimal system that may interfere with functional and cosmetic success [15, 16].

Jordan described current techniques for MCT laxity repair as technically difficult and do not always achieve a satisfactory outcome. These techniques only aim at firm medial fixation with little consideration to fine anatomical and physiological details of the medial canthus. Due to the hazards associated with these procedures, most surgeons avoided early MCT repair, and surgery may be delayed until the MCT laxity become advanced [16].

In this study, we described the effect of double-frequency YAG laser applied to lower medial palpebral conjunctiva in early cases with grade ≤ 2 MCT laxity. This technique aims to create a firm conjunctival scar below the lower punctum, sparing the canaliculus to reverse everted punctum to normal position.

We used an objective measurement for evaluation correction of punctual eversion in the form of decrease of tear film meniscus measured by imageJ software. We also correlate the objective data in the form of tear film meniscus level with patient satisfaction after amelioration of epiphora. It is considered a unique method for assessment as none of the previous literature mentioned objective documentation of resolution of symptoms after correction of ectropion.

There was a highly significant difference between values recorded before and after double frequency YAG laser intervention regarding the height of tear film which was found to be significantly lower after the intervention compared to before it (81.1 versus 193.1, respectively). The more the degree of reduction of tear film height, the more the satisfaction of patients.

To our knowledge, using of argon laser without excision of tissue, or the use of full-thickness everting sutures has been described only at 2003 by Nainiwal S, and co-authors [17] and achieved good functional results by relief of symptoms in 22 (73%) eyes, and an acceptable anatomical outcome with repositioning of the punctum in 24 (80%) eyes.

In their retrospective consecutive case series performed over 6 years in medial ectropion with moderate to severe medial canthal tendon laxity, Vahdani K and Thaller VT described medial canthal thermoplasty (MCT) as an adjunctive procedure to standard surgery for correcting their eyelid malposition that included lateral Bick’s shortening ± medial retractor plication. Strong diathermy was applied with bipolar forceps, avoiding the canaliculus to whiten the conjunctiva and underlying Horner’s muscle. They concluded that MCT only stabilizes the medial canthus in treated cases, so it must be combined with an eyelid shortening procedure if significant laxity persists [9].

Medial lower eyelid ectropion with mild to moderate degree of medial canthal tendon laxity is corrected with horizontal eyelid shortening procedures such as retro-punctal cautery, lazy-T procedure, medial spindle, and resection of the posterior lamellar flap. These procedures can be augmented by horizontal eyelid tightening using a lateral tarsal strip or a full-thickness pentagonal resection [18].

Medial spindle surgery involves the excision of a diamond-shaped part of the conjunctiva and retractors with the sutures tied anteriorly on the skin [19]. The lazy-T technique, described by Byron Smith in 1976, is another option for the treatment of medial ectropion of the lower lid. Both Horizontal and vertical eyelid shortening is achieved by full-thickness excision of a portion of the lower lid as well as the posterior lamella in a sideways [20]. Both of these procedures are invasive and involve the excision of part of posterior lamella tissue to achieve inversion of the eyelid. However, the procedure described in our study is less invasive and can achieve the same effect.

Goel and colleges noted that at 1-year follow-up, anatomical success was achieved in 28 (90%) patients and functional success in the form of the disappearance of epiphora was noted in 27 (87%) patients after lower eyelid suspension using polypropylene suture for the correction of punctal ectropion. They also mentioned that results did not correlate to the type of laxity nor the degree of ectropion [21]. Raus and colleges concluded that repair of early to intermediate ectropion of the lacrimal punctum using the Raus–Garito clamp has a good functional and cosmetic outcome [22].

Double frequency YAG laser on the lower medial palpebral conjunctiva has many advantages. It is a simple, easy, and quick technique that can be performed under topical anesthesia in the outpatient clinic. Furthermore, it is minimally invasive (non-incisional) and avoids excision of any part of the posterior lamella or conjunctiva. Unlike medial canthal resection procedures, it is safe for the canalicular and the lacrimal system with no risk of trauma or injury.

Limitations of the current study were the small number of cases due to inclusion of only patients with no or early MCT laxity and short follow-up period. Advanced cases with punctal eversion more than grade 2 were not included in the study. Further studies including these cases with more patients and a longer duration of follow-up are needed.

## Conclusion

Double-frequency YAG laser applied to the lower medial palpebral conjunctiva is a simple, easy, safe, and effective procedure that can be used as a solo treatment in early cases of punctal eversion with no or mild medial canthal tendon laxity. It has good anatomical and functional outcomes, preserves the canaliculus, and eliminates or reduces the need for eyelid resection. It gives satisfactory relief of symptoms, with minimal or no complications.

## Data Availability

Data available on request from the authors.

## Declarations

### Animal research

The study was not conducted on animals.

## References

[1] Fong KC, Mavrikakis I, Sagili S (2006). Correction of involutional lower eyelid medial ectropion with transconjunctival approach retractor plication and lateral tarsal strip. Acta Ophthalmol Scand.

[2] Lee H, Hwang J-Y, Kim JW (2013). The effectiveness of a simultaneous medial spindle procedure for involutional punctal ectropion with lid laxity in patients who require endonasal dacryocystorhinostomy instead of external dacryocystorhinostomy to prevent pump failure. J Craniofac Surg.

[3] Mitchell DA, Lyons AB, Moy RL (2018). Correction of cicatricial and involutional lower eyelid ectropion with hyaluronic acid. JAAD Case Rep.

[4] Goel R, Sanoria A, Kumar S (2017). Comparison of polypropylene sling with combined transconjunctival retractor plication and lateral tarsal strip for correction of involutional lower eye lid ectropion. Open Ophthalmol J.

[5] Guthrie AJ, Kadakia P, Rosenberg J (2019). Eyelid malposition repair: a review of the literature and current techniques. Semin Plast Surg.

[6] Kam K, Cole C, Bunce C (2012). The lateral tarsal strip in ectropion surgery: is it effective when performed in isolation?. Eye.

[7] Liu CY, Oh DJ, Putterman AM (2019). A lazy-T Modification in the treatment of medial ectropion. Aesthet Surg J.

[8] O'Donnell B (1994). Age-related medial ectropion of the lower eyelid. Aust N Z J Ophthalmol.

[9] Vahdani K, Thaller VT (2017). Posterior medial canthal thermoplasty. Ophthalmic Plast Reconstr Surg.

[10] Bandlitz S, Pult H (2016). Advances in tear film assessment. Optom Pract.

[11] Simoes P, Silva P, Cordeiro M (2018). Repeatability and reproducibility of peripapillary choroidal thickness using a medical image-processing software. Med Hypothesis, Discov Innov Ophthalmol.

[12] Rodrigues R, Dos Santos MS, Silver RE (2019). Corneal incision architecture: VICTUS femtosecond laser vs manual keratome. Clin Ophthalmol (Auckl NZ).

[13] Olver JM, Sathia PJ, Wright M (2001). Lower eyelid medial canthal tendon laxity grading: an interobserver study of normal subjects. Ophthalmology.

[14] Liebau J, Schulz A, Arens A (2006). Management of lower lid ectropion. Dermatol Surg.

[15] Clement CI, O'Donnell BA (2004). Medial canthal tendon repair for moderate to severe tendon laxity. Clin Exp Ophthalmol.

[16] O’Donnell B, Anderson R, Collin J (2003). Repair of the lax medial canthal tendon. Br J Ophthalmol.

[17] Nainiwal S, Kumar H, Kumar A (2003). Laser conjunctivoplasty: a new technique for correction of mild medial ectropion. Orbit.

[18] Ibrahim HA, Sabry HN (2014). Classification and management of ectropion with medial canthal tendon laxity. J Egypt Ophthalmol Soc.

[19] Nowinski TS, Anderson RL (1985). The medial spindle procedure for involutional medial ectropion. Arch Ophthalmol.

[20] Smith B (1976). The lazy-T correction of ectropion of the lower punctum. Arch Ophthalmol.

[21] Goel R, Kamal S, Bodh SA (2013). Lower eyelid suspension using polypropylene suture for the correction of punctal ectropion. J Cranio-Maxillofac Surg.

[22] Raus PP, Bral N, Collin R (2018). Punctal Ectropion repair using the Raus-Garito clamp. Orbit.

